# Time to deterioration of patient-reported outcomes in non-small cell lung cancer: exploring different definitions

**DOI:** 10.1007/s11136-022-03088-0

**Published:** 2022-01-31

**Authors:** Andrew Walding, Konstantina Skaltsa, Montserrat Casamayor, Anna Rydén

**Affiliations:** 1 AstraZeneca R&D, 132 Hills Road, Cambridge, CB2 1PG UK; 2 IQVIA, Provença 392, 3rd floor, 08025 Barcelona, Spain; 3 AstraZeneca Gothenburg, Pepparedsleden 1, 431 83 Mölndal, Sweden

**Keywords:** Disease progression, Non-small cell lung cancer, Patient-reported outcomes

## Abstract

**Purpose:**

The clinical relevance of different time-to-deterioration **(**TTD) definitions for patient-reported outcomes were explored.

**Methods:**

TTD definitions differing by reference score and deterioration event were used to analyse data from the phase 3 FLAURA trial of first-line osimertinib versus erlotinib or gefitinib in patients with EGFR-mutated advanced non-small cell lung cancer. Pre-specified key symptoms were fatigue, appetite loss, cough, chest pain and dyspnoea, scored using the European Organisation for Research and Treatment of Cancer QLQ-C30 and QLQ-LC13 questionnaires (≥ 10-point difference = clinically relevant).

**Results:**

No significant treatment differences in TTD (distributions) were observed using definitions based on transient or definitive deterioration alone. TTD definitions based on definitive, sustained deterioration, with death not included as an event, yielded a significant treatment difference for dyspnoea (hazard ratio [HR] 0.71; *P* = 0.034) when baseline was the reference, and for cough (HR 0.70; *P* = 0.009) and dyspnoea (HR 0.71; *P* = 0.004) when best previous score was the reference. With death included as an event, treatment differences were significant for dyspnoea (HR 0.70; *P* = 0.025) when baseline was the reference, and for cough (HR 0.70; *P* = 0.011), dyspnoea (HR 0.71; *P* = 0.003) and chest pain (HR 0.71; *P* = 0.038) when best previous score was the reference. Irrespective of definition, TTD for appetite loss and fatigue did not differ significantly between arms.

**Conclusion:**

This exploratory work showed that different TTD definitions yield different magnitudes of treatment difference, highlighting the importance of pre-specifying TTD definitions upfront in clinical trials.

**Clinical trial registration:**

ClinicalTrials.gov NCT02296125.

**Supplementary Information:**

The online version contains supplementary material available at 10.1007/s11136-022-03088-0.

## Introduction

Patient-reported outcomes (PROs) are incorporated as endpoints in clinical trials to assess the clinical benefit of new treatments for patients [1, 2]. The most widely used instruments in phase 3 oncology trials are the European Organisation for Research and Treatment of Cancer (EORTC) questionnaires [2]. PRO-derived data, such as those obtained from EORTC questionnaires, complement standard overall survival (OS) or tumour-based efficacy endpoints [3]. In addition to group-level analysis for mean-change-from-baseline-type endpoints, PRO data can be assessed longitudinally at the level of individual patient ‘events’ using time-to-deterioration (TTD) analysis [2, 4]. Use of the TTD approach relies on a clear definition of deterioration event characteristics, such as the reference score relative to which the deterioration is quantified, the deterioration and censoring event definition, and the within-patient score change threshold that defines a clinically relevant deterioration.

TTD definitions are not standardised and vary across oncology trials [2]. The TTD definition, analysis and study design need to align with the clinical question posed, and the method used for handling intercurrent events (e.g. death, disease progression) needs to be outlined upfront [5, 6]. Anota and colleagues proposed standardising TTD analysis methods for PROs to be used as endpoints together with tumour parameters such as progression-free survival (PFS) [1]. Several different definitions of TTD were described, and standardisation was proposed according to the therapeutic setting [1]. The EORTC’s Setting International Standards for the Analysis of Quality of Life (SISAQOL) consortium is working on standardising PRO analysis, with an initial oncology focus [7]. Efforts are ongoing, including on statistical methods, standardization of statistical terms and management of missing data [7]. The SISAQOL consortium agreed that clinical relevance is an essential criterion for PRO interpretation [7].

In the FLAURA trial, the third-generation epidermal growth factor receptor (EGFR) tyrosine kinase inhibitor osimertinib prolonged PFS and OS compared with erlotinib or gefitinib as first-line therapy in patients with *EGFR*-mutated advanced non-small cell lung cancer (NSCLC) [8]. TTD analysis of key symptoms, defined as first transient deterioration versus baseline, found no clinically meaningful difference between treatment arms [9].

The aim of the current work was to explore the clinical relevance of different definitions of TTD for PROs to understand better whether the TTD definition used in the FLAURA PRO analysis contributed to the apparent discrepancy between PFS and OS results compared with PRO data. This work was exploratory and was not intended as a basis of evidence of clinical benefit.

## Materials and methods

### Source data

Data used for this work were from FLAURA (ClinicalTrials.gov identifier: NCT02296125), a multinational, double-blind, randomised phase 3 trial [8]. Patients enrolled in FLAURA had locally advanced or metastatic NSCLC with an *EGFR* mutation (exon 19 deletion or L858R) and were eligible to receive first-line treatment with erlotinib or gefitinib. Patients were stratified by tumour *EGFR* mutation status (Ex19del or L858R) and race (Asian or non-Asian), and were randomised 1:1 to receive study treatment (oral osimertinib 80 mg once daily; *n* = 279) or an active comparator (oral erlotinib 150 mg once daily or oral gefitinib 250 mg once daily, as pre-specified by each participating study site; *n* = 277). All patients were followed up for PFS and OS every 6 weeks until the primary cut-off date (June 2017). The FLAURA trial was conducted in accordance with the Declaration of Helsinki. All patients provided written informed consent.

### PRO assessments

PROs were assessed prospectively in the FLAURA trial using the EORTC Quality of Life Questionnaire Core 30 items (QLQ-C30) and Lung Cancer 13 items (QLQ-LC13) [9–11]. Patients completed the QLQ-C30 at baseline and every 6 weeks thereafter, and the QLQ-LC13 at baseline, then weekly for the first 6 weeks and every 3 weeks thereafter, until secondary radiographic disease progression was documented. Exploratory FLAURA TTD analyses were conducted for the protocol pre-specified key symptoms fatigue (three items; QLQ-C30), appetite loss (one item; QLQ-C30), cough (one item; QLQ-LC13), chest pain (one item; QLQ-LC13) and dyspnoea (three items; QLQ-LC13) [9]. Raw scores were converted to standardised scores from 0 to 100 [10, 11]. Higher scores represent more/worse symptoms. A 10-point within-patient score change was the threshold used to define a clinically relevant change [9].

### Exploratory analyses

Six TTD definitions were considered for assessing treatment differences in TTD prior to radiographic disease progression or death (Table 1; Supplementary Table S1). Definitions were developed based on those described by Anota and colleagues [1], and explored different requirements for the deterioration event (transient, definitive, sustained) and reference score (baseline [means shown in Supplementary Table S2], best previous), as follows:

**Table 1:** TTD endpoint definitions^a^

Name	Definition
TTD1 (transient)	• Time to first *deterioration* of ≥ 10 points versus *best previous score*^b^
	• Patients with no deterioration before end of follow-up, radiographic disease progression or death to be censored at last available PRO assessment^c^
TTD2 (transient, confirmed at subsequent visit)	• Time to first *deterioration* of ≥ 10 points versus *baseline score confirmed at a subsequent assessment* ≥ *3 weeks* (21 days) later (or sustained for 3 weeks)
	• Patients with no confirmed deterioration before end of follow-up, radiographic disease progression or death^d^ to be censored at last available PRO assessment^c^
TTD3 (definitive)	• Time to first *definitive deterioration with no subsequent clinically meaningful improvement* defined as time to first deterioration of ≥ 10 points versus *baseline score* and no subsequent improvement of ≥ 10 points versus baseline score before the end of follow-up, radiographic disease progression or death^d^
	• Patients with no definitive deterioration before end of follow-up, radiographic disease progression or death to be censored at the last available PRO assessment^c^
TTD4 (definitive, sustained)	• Time to first *definitive deterioration* of ≥ 10 points versus *baseline score* and a *sustained deterioration of* ≥ *10 points* versus baseline score at all subsequent time points before end of follow-up, radiographic disease progression or death^d^
	• Patients with no definitive deterioration before end of follow-up, radiographic disease progression, or death to be censored at the last available PRO assessment^c^
TTD5 (definitive)	• Time to first *definitive deterioration with no subsequent clinically meaningful improvement* defined as time to first deterioration of ≥ 10 points versus *best previous score*^b^ and no subsequent improvement of ≥ 10 points versus best previous score before end of follow-up, radiographic disease progression or death^d^
	• Patients with no definitive deterioration before end of follow-up, radiographic disease progression or death to be censored at the last available PRO assessment^c^
TTD6 (definitive, sustained)	• Time to first *definitive deterioration* of ≥ 10 points versus *best previous score*^b^ and a *sustained deterioration of* ≥ *10 points* versus best previous score at all subsequent time points before end of follow-up, radiographic disease progression or death^d^
	• Patients with no definitive deterioration before end of follow-up, radiographic disease progression or death to be censored at the last available PRO assessment^c^

^a^Separate analyses were performed with death not included and included as an event

^b^Best previous score can be baseline or post baseline

^c^Last available PRO assessment for the corresponding item/scale

^d^If no radiographic disease progression before death

TTD1 (transient vs. best previous)—the time to the first deterioration of at least 10 points compared to the best previous score;

TTD2 (transient confirmed vs. baseline)—the time to the first deterioration of at least 10 points compared to the baseline score confirmed at a subsequent assessment at least 3 weeks later;

TTD3 (definitive vs. baseline)—the time to the first deterioration of at least 10 points compared to the baseline score and with no subsequent improvement of 10 points or more compared to the baseline score;

TTD4 (definitive sustained vs. baseline)—the time to the first deterioration of at least 10 points compared to the baseline score, sustained at all subsequent time points;

TTD5 (definitive vs. best previous)—the time to the first deterioration of at least 10 points compared to best previous score and no subsequent improvement of 10 points or more compared to the best previous score;

TTD6 (definitive sustained vs. best previous)—the time to the first deterioration of at least 10 points compared to the best previous score, sustained at all subsequent time points.

First transient deterioration compared with baseline, the definition used in the FLAURA PRO analysis [9], was not reassessed.

The full analysis set included all randomly assigned patients in the FLAURA trial. TTD was estimated in the full analysis set using Kaplan–Meier methodology and the log-rank test. Patients with baseline scores above 90 were censored on day 1. Patients not experiencing deterioration events and those who discontinued participation in the trial were censored at their last PRO assessment. Intermittent missing data were treated as missing at random and were not considered a deterioration. Monotone missing data occurring because of disease progression, treatment discontinuation or death were handled using the censoring/event rules as per the TTD definition being assessed. *P* values were obtained from a stratified log-rank test with the stratification variables mutation type (Ex19del, L858R) and race (Asian, non-Asian), using the Breslow approach for handling ties. *P* values < 0.05 were considered to be statistically significant, and values between 0.05 and 0.10 were considered to be trending towards statistical significance. Hazard ratios (HRs) with 95% confidence intervals (CIs) were obtained from a Cox proportional hazards model, using the Efron approach for handling ties [12], with treatment, baseline score and baseline central nervous system metastasis status as covariates, and race and mutation type as stratification variables. Separate analyses were performed with death not included as an event (patients censored at death) and death included as an event, when death occurred within one assessment window after the last available PRO assessment.

Additional exploratory analyses were performed to explore the nature and timing of symptom worsening. Slopes of PRO scores over time were evaluated before radiographically progressed disease or death for patients with disease progression or patients who died, using a mixed model for repeated measures (MMRM) with continuous time effect (study day). Observed mean PRO values over time were summarised graphically. The Adaptive SIDEScreen method was used to examine baseline characteristics potentially predictive of enhanced treatment effect in terms of time to PRO deterioration before radiographically progressed disease or death [13]. The baseline characteristics assessed were age, sex, body mass index, extent of disease (metastatic, locally advanced, both), site of local/metastatic disease, brain metastases and visceral metastases, tumour size, histology type, smoking status, World Health Organization performance status, and QLQ-C30 and QLQ-LC13 scores. Descriptive graphical summaries were produced for mean values of PRO scores over time distinguishing assessments before and after disease progression, by treatment group. The plots were also produced separately for the subset of patients who did not progress.

## Results

### TTD treatment differences

**Fig. 1:**
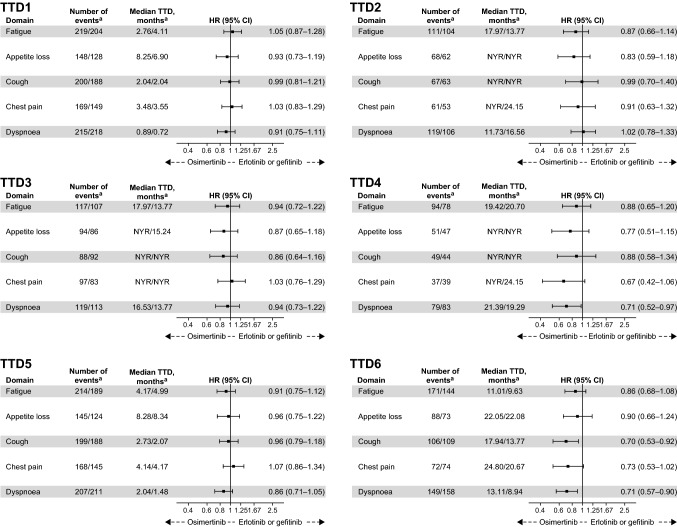
TTD analysis of pre-specified key symptoms for six different TTD definitions assessed using Kaplan–Meier estimates, with death not counted as an event. HRs with 95% CIs were calculated using a stratified Cox regression model with the stratification variables mutation type and race, and the covariates treatment, baseline score and baseline central nervous system metastasis status. ^a^Osimertinib (*n* = 279)/gefitinib or erlotinib (*n* = 277)

Results for treatment differences in TTD of the five pre-specified key symptoms, using the six different TTD definitions, are shown in Fig. 1 with death not included as an event and in Supplementary Fig. S1 with death included as an event.

No significant treatment differences in TTD (distributions) were observed using definitions based on transient deterioration events (TTD1) or confirmed deterioration events at two subsequent visits (TTD2), irrespective of whether death was or was not included as an event. The two definitions based on definitive deterioration alone (i.e. not sustained; TTD3, TTD5) similarly did not yield significant treatment differences, irrespective of whether death was or was not included as an event.

The two TTD definitions based on definitive, sustained deterioration (TTD4, TTD6) resulted in significant treatment differences. With death not included as an event, definition TTD4 (i.e. reference score = baseline) yielded a significant treatment difference for dyspnoea (HR 0.71; *P* = 0.034) and a trend towards significance for chest pain (HR 0.67; *P* = 0.086); and definition TTD6 (i.e. reference score = best previous) yielded significant differences for cough (HR 0.70; *P* = 0.009) and dyspnoea (HR 0.71; *P* = 0.004) and a trend towards significance for chest pain (HR 0.73; *P* = 0.067). With death included as an event, definition TTD4 yielded a significant difference for dyspnoea (HR 0.70; *P* = 0.025) and a trend towards significance for chest pain (HR 0.65; *P* = 0.054); and definition TTD6 yielded significant differences for cough (HR 0.70; *P* = 0.011), dyspnoea (HR 0.71; *P* = 0.003) and chest pain (HR 0.71; *P* = 0.038).

Whatever the definition applied, the results using TTD for appetite loss and fatigue did not differ significantly between treatment arms. Owing to a lower frequency of assessment of appetite loss and fatigue there was reduced power in detecting an effect for these symptoms than for cough, dyspnoea and chest pain.

### PRO scores over time

**Fig. 2:**
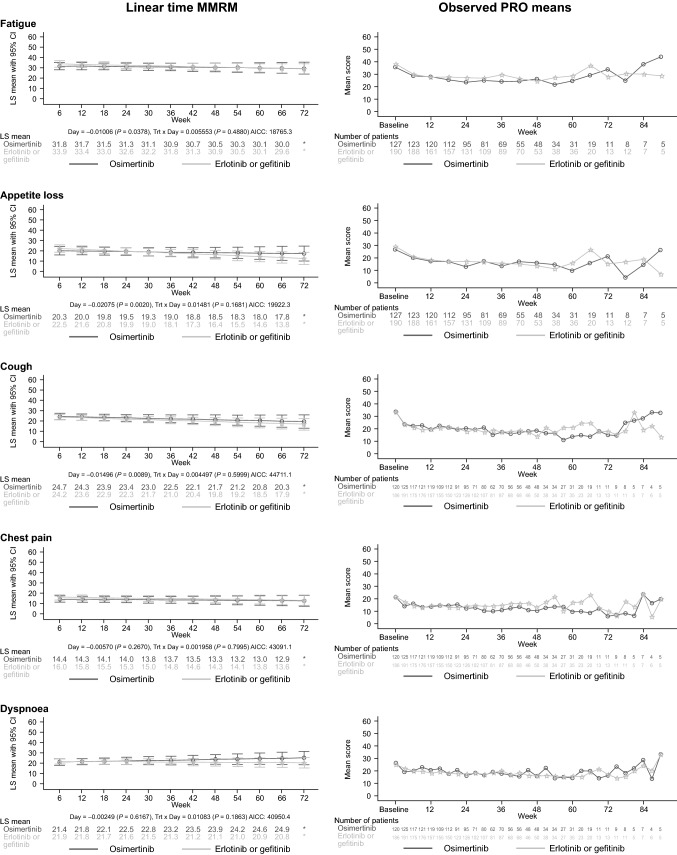
Linear-time MMRM (left panels) and observed means (right panels) for PROs before radiographically progressed disease or death in patients who had radiographic disease progression or who died

Linear-time MMRM and observed PRO means over time before radiographically progressed disease or death are shown in Fig. 2 for patients whose disease progressed or who died. No statistically significant between-treatment differences in MMRM slopes were observed. In both treatment groups, MMRM slopes over time were non-zero in the direction of PRO improvement for fatigue, appetite loss and cough, and close to zero for chest pain and dyspnoea. Observed PRO means suggested similar symptom trajectories for both treatment groups. Generally, some improvement was seen during approximately the first 12 weeks from baseline, followed by an apparent stabilisation in the absence of radiographic disease progression. Some worsening and much variability was observed at later time points, particularly after 60 weeks, when numbers of patients with available data were considerably reduced.

In patients with radiographic disease progression, fatigue, appetite loss and cough were generally worse after disease progression than before it in both treatment groups, and for fatigue and appetite loss, values after disease progression appeared worse in the erlotinib or gefitinib arm than in the osimertinib arm (Supplementary Fig. S2). Compared with patients with disease progression, those without disease progression generally had better values over time for fatigue, appetite loss and cough, and for cough after 48 weeks values appeared better in the osimertinib arm than in the erlotinib or gefitinib arm. For dyspnoea and chest pain, values were similar regardless of disease progression.

### Baseline characteristics as predictive factors

No baseline characteristic assessed was a strong predictor of enhanced osimertinib treatment effect in terms of TTD. Female sex was a weak predictor of increased TTD of fatigue when using definition TTD6 (treatment effect in full data set, HR 0.867; in the female cohort, HR 0.769).

## Discussion

TTD is an increasingly used PRO endpoint in oncology clinical trials and the TTD format of presenting trial results is familiar to clinicians [1, 2]. Although efforts to consolidate analysis and reporting of longitudinal PRO data, led by the SISAQOL consortium, are ongoing [7], TTD definitions and methodological approaches are not presently standardised [1, 2]. The current analysis in the advanced NSCLC setting explored different TTD definitions using data from the phase 3 FLAURA trial [8]. Marked treatment differences were observed for the key NSCLC symptoms of cough, dyspnoea and chest pain using TTD definitions based on definitive, sustained deterioration but not those based on transient, confirmed or definitive deterioration alone. No significant treatment differences were observed for appetite loss and fatigue, irrespective of the TTD definition used. Discrepancies may be due to the timings of PRO and radiographic assessments and the exclusion of post-progression PRO values from the TTD definition, and to appetite loss and fatigue potentially being more likely than other key symptoms to overlap with treatment-related symptoms. Additionally, appetite loss and fatigue were assessed less frequently than cough, dyspnoea and chest pain.

The most appropriate TTD definition will vary depending on stakeholder needs, and the clinical question of interest. Handling of the intercurrent events of disease progression or death needs to be outlined at the time of defining the estimand for the PRO time-to-event endpoint [14]. For example, if progression should be ‘penalised’, then a composite strategy of considering progression as an event should be employed. If a ‘while not progressed’ approach is to be followed, then patients are censored at progression or alternative frameworks may be warranted (e.g. competing risks). Similar considerations can be made for death. For progression specifically, another option may be the treatment policy strategy that is thought of as the closest to the intent-to-treat principle, which aims to quantify the treatment effect regardless of progression, that is, post-progression PRO data should be collected and included in the analysis. The target population, expected timings of events of interest and duration of deterioration will need to be considered. For example, cough may be expected to deteriorate before disease progression, whereas cognitive functioning may not deteriorate until well after progression. In the advanced cancer setting, time until definitive deterioration may be more appropriate than time until any (transient) deterioration to reflect health deterioration caused by the underlying disease course [4]. For time to first deterioration, the choice between transient and definitive deterioration will depend on whether transient changes are part of the focus of the investigation. For example, in the initial days or weeks of a clinical trial, short-term adverse events can lead to a transient, reversible worsening in symptoms and health-related quality of life (HRQoL) and quantifying a transient deterioration may favour one treatment arm, but may not be of interest to the regulator if it is thought that it will resolve and the conceptual model indicates it is of no important impact for the patient. The present results were affected by the choice of reference score. For definitive, sustained deterioration, significant between-treatment differences were observed for cough when the reference score was the best previous score but not the baseline score, whereas for dyspnoea treatment differences were significant regardless of reference score. Considering baseline as the reference score may be appropriate in the asymptomatic disease setting but could lead to meaningful deterioration being missed in settings where symptom improvement is expected after treatment start. Use of best previous score as the reference can take response shift into account [1, 4]. The choice of score change threshold considered clinically relevant may have an impact on the sensitivity of the results. The 10-point within-patient score change used in the FLAURA trial EORTC analysis is the most frequently used threshold in pivotal oncology clinical trials [15], usually claimed to be informed by the work of Osoba et al. [16], although Osoba et al.’s work never attempted to provide estimates for all EORTC domains. Thresholds specific to therapeutic settings and cancer site are being assessed [17–19].

In the FLAURA trial, although patients were being treated in the first-line setting, they had advanced lung cancer, with 95% having metastatic disease [8]. Ignoring death in the TTD definition in advanced cancer could represent informative censoring [2], that is, censoring at death assumes that patients who died have the same probability of deteriorating as patients who are alive. In the present work, when death was included as an event a time constraint was added to incorporate deaths that were close to the time of PRO assessments and were thus likely to be relevant to the deterioration evaluation, and to avoid inclusion of long-term events occurring after a long period with no PRO information. Including death as an event did not substantially alter the definitive, sustained deterioration results from those obtained not including death as an event when baseline was the reference; however, it strengthened the statistical significance of the treatment difference for chest pain when best previous score was the reference. For intercurrent events of radiographic disease progression or death, the current work employed a ‘while on treatment’ strategy, which discards data after intercurrent events and typically treats intermittent missing data as missing at random. This differs from a treatment policy strategy, which would include information reported after the intercurrent event and require a sensitivity analysis.

The US Food and Drug Administration considers time to progression of cancer symptoms a direct measure of clinical benefit rather than a potential surrogate endpoint [20]. Disease progression has been shown to be associated with worsening HRQoL in patients with solid, metastatic cancers [21]. In the current analysis, fatigue, appetite loss and cough were generally worse after disease progression than before it commenced. Some patients with radiographic disease progression may not have symptom deterioration, especially if the overall symptom burden is low, and PRO questionnaires may not detect symptom deterioration that does occur. Symptom deterioration patterns can depend on the symptom being assessed, and the type, extent and site of progression. For example, progression may result in a new symptom not being measured in the TTD analysis, progression at several sites may result in faster deterioration than progression at a single site, and progression at the lung’s periphery may result in more chest pain than progression at its centre.

The current work had important strengths. Data used were from a double-blind, randomised trial. Methods employed were consistent with recent SISAQOL guidelines [7]. Questionnaire completion rates were high, with more than 70% of patients in both treatment arms (full analysis set) who were expected to complete their PRO questionnaires completing both the EORTC QLQ-C30 and QLQ-LC13 at most time points (see Supplementary appendix in [9]). PRO data were collected frequently during the study. Limitations include the fact that the analysis focused on symptom burden and did not specifically cover functioning, that it was conducted in one setting only and that only one score change threshold was assessed. Adherence to treatment and anticipated event rate need to provide confidence in the TTD definition selected for primary analysis. Future researchers may wish to explore TTD definitions in early cancer interventions and evaluate the effect of an open-label data set on analyses. The current research adds to the broader efforts to advance and standardise PRO analysis in oncology trials.

## Conclusions

If TTD is the endpoint being considered, the event of interest and strategies to handle intercurrent events should be well defined upfront within the context of use. Our exploratory analyses demonstrate that TTD results differ depending on the clinical questions being asked. Thoughtful consideration will lead to the most clinically relevant questions and corresponding estimands being decided upfront. The most appropriate TTD definition may vary depending on the needs of different stakeholders. Predefining more than one definition may improve our understanding of the impact of different interventions on TTD.

## Supplementary Information

Below is the link to the electronic supplementary material.

Supplementary file1 (DOCX 428 KB)

## Data Availability

All data generated during this analysis are included in the article and its supplementary files.
