# Correction to: Time to deterioration of patient-reported outcomes in non-small cell lung cancer: exploring different definitions

**DOI:** 10.1007/s11136-022-03128-9

**Published:** 2022-03-24

**Authors:** Andrew Walding, Konstantina Skaltsa, Montserrat Casamayor, Anna Rydén

**Affiliations:** 1 AstraZeneca R&D, 132 Hills Road, Cambridge, CB2 1PG UK; 2 IQVIA, Provença 392, 3rd floor, 08025 Barcelona, Spain; 3 AstraZeneca Gothenburg, Pepparedsleden 1, 431 83 Mölndal, Sweden

## Correction to: Quality of Life Research 10.1007/s11136-022-03088-0

The article “Time to deterioration of patient‑reported outcomes in non‑small cell lung cancer: exploring different definitions”, written by Andrew Walding, Konstantina Skaltsa, Montserrat Casamayor and Anna Rydén, was originally published electronically on the publisher’s internet portal on 31 January 2022 without open access. With the author(s)’ decision to opt for Open Choice the copyright of the article changed on 15 March 2022 to © The Author(s) 2022 and the article is forthwith distributed under a Creative Commons Attribution 4.0 International License, which permits use, sharing, adaptation, distribution and reproduction in any medium or format, as long as you give appropriate credit to the original author(s) and the source, provide a link to the Creative Commons licence, and indicate if changes were made. The images or other third party material in this article are included in the article’s Creative Commons licence, unless indicated otherwise in a credit line to the material. If material is not included in the article’s Creative Commons licence and your intended use is not permitted by statutory regulation or exceeds the permitted use, you will need to obtain permission directly from the copyright holder. To view a copy of this licence, visit http://creativecommons.org/licenses/by/4.0.

The original article has been corrected

