# Supplementary material for: Time to deterioration of patient-reported outcomes in non-small cell lung cancer: exploring different definitions
Source: Qual Life Res. 2022 Jan 31;31(8):2535–43. doi: 10.1007/s11136-022-03088-0 (PMC9250481; doi:10.1007/s11136-022-03088-0)
Supplement: Supplementary file 1 — Supplementary file1 (DOCX 428 KB) [file 11136_2022_3088_MOESM1_ESM.docx]

**Supplementary Information**

**Time to deterioration of patient-reported outcomes in non-small cell lung cancer: which definition is clinically relevant?**

*Qual Life Res*

Andrew Walding^1,^*, Konstantina Skaltsa^2^, Montserrat Casamayor^2^, Anna Rydén^3^

^1^ *AstraZeneca R&D, 132 Hills Road, Cambridge CB2 1PG, UK*

^2^ *IQVIA, Provença 392, 3rd floor, 08025 Barcelona, Spain*

^3^ *AstraZeneca Gothenburg, Pepparedsleden 1, SE-431 83, Mölndal, Sweden*

***** *Corresponding author*: AstraZeneca R&D, 132 Hills Road, Cambridge CB2 1PG, UK.

*E-mail address*: [andrew.walding@astrazeneca.com](mailto:andrew.walding@astrazeneca.com) (A. Walding).

**Supplementary Table S1** Summary of the different TTD definitions

|  | TTD1 | TTD2 | TTD3 | TTD4 | TTD5 | TTD6 |
| --- | --- | --- | --- | --- | --- | --- |
| **Type of deterioration** |  |  |  |  |  |  |
| Transient | X | X |  |  |  |  |
| Definitive |  |  | X | X | X | X |
| Confirmed |  | X |  |  |  |  |
| Sustained |  |  |  | X |  | X |
| **Reference** |  |  |  |  |  |  |
| Baseline |  | X | X | X |  |  |
| Best previous score | X |  |  |  | X | X |

TTD, time-to-deterioration

**Supplementary Table S2** Mean (standard deviation) EORTC QLQ-C13 and QLQ-C30 scores of pre-specified key symptoms at baseline

|  | Osimertinib | Erlotinib/gefitinib |
| --- | --- | --- |
| **QLQ-LC13** |  |  |
| Cough | 32.8 (27.2) | 33.5 (28.8) |
| Dyspnoea | 22.5 (23.1) | 25.0 (22.8) |
| Chest pain | 19.5 (25.3) | 20.8 (25.7) |
| **QLQ-C30** |  |  |
| Fatigue | 32.2 (24.9) | 35.8 (26.2) |
| Appetite loss | 22.7 (28.5) | 25.6 (29.9) |

EORTC, European Organisation for Research and Treatment of Cancer; QLQ-C30, Quality of Life Questionnaire Core 30 items; QLQ-LC13, Quality of Life Questionnaire Lung Cancer 13 items

**Supplementary Fig. S1** TTD analysis of pre-specified key symptoms for the six different TTD definitions, assessed using Kaplan–Meier estimates, with death counted as an event. HRs with 95% CIs were calculated using a stratified Cox regression model with the stratification variables mutation type and race, and the covariates treatment, baseline score and baseline central nervous system metastasis status. ^a^ Osimertinib (n = 279)/gefitinib or erlotinib (n = 277)

TTD, time-to-deterioration

**Supplementary Fig. S2** Observed means for PROs over time in patients with (left panels) and without (right panels) radiographic disease progression

PRO, patient-reported outcome
